# Bridging the gaps: the gut-lung axis and microbial metabolites in the pathogenesis and treatment of pulmonary fibrosis

**DOI:** 10.3389/fmed.2026.1817835

**Published:** 2026-04-30

**Authors:** Yingxu Wu, Hao Yan, Pin Li, Yongming Liu, Jiapeng Leng, Yuwei Cui, Xiaodong Lv, Lijian Pang, Ningzi Zang

**Affiliations:** 1 First Clinical College, Liaoning University of Traditional Chinese Medicine, Shenyang, Liaoning, China; 2 Science and Technology Management Department, Affiliated Hospital of Liaoning University of Traditional Chinese Medicine, Shenyang, Liaoning, China; 3 Department of Traditional Chinese Medicine Experimental Center, Affiliated Hospital of Liaoning University of Traditional Chinese Medicine, Shenyang, Liaoning, China; 4 College of Pharmacy, Liaoning University of Traditional Chinese Medicine, Dalian, Liaoning, China; 5 Department of Respiratory, Affiliated Hospital of Liaoning University of Traditional Chinese Medicine, Shenyang, Liaoning, China

**Keywords:** gut microbiome, gut microbiota, gut-lung axis, microbial metabolites, pulmonary fibrosis

## Abstract

Pulmonary fibrosis (PF) is a chronic interstitial lung disease characterized by structural damage to the lung parenchyma, excessive deposition of extracellular matrix (ECM), and irreversible decline in lung function. Current pharmacological treatments cannot effectively reverse fibrosis, highlighting an urgent need for novel therapeutic targets. Recently, the gut-lung axis and its bidirectional communication have received increasing attention for their roles in PF progression. Metabolites derived from gut microbiota, including short-chain fatty acids (SCFAs), bile acids, tryptophan metabolites, lipopolysaccharides (LPS), and trimethylamine N-oxide, regulate immune responses, modulate signaling pathways, influence epigenetic modifications, and maintain intestinal barrier integrity, thereby exerting bidirectional effects on PF. Protective metabolites primarily inhibit fibroblast activation and collagen deposition, whereas pathological metabolites promote fibrosis by inducing inflammatory responses and oxidative stress. Potential therapeutic strategies targeting the gut-lung axis include fecal microbiota transplantation (FMT), probiotic and dietary interventions, and Traditional Chinese Medicine (TCM). However, clinical applications face challenges such as donor standardization, immunological safety, and consistency of therapeutic efficacy. Critical limitations remain, including reliance on acute-injury animal models that inadequately represent the chronic, irreversible nature of human PF. Translating findings across distinct PF subtypes requires caution, as their genetic architectures, immune landscapes, and microbiome interactions may differ considerably. Additionally, the causal relationship between microbial dysbiosis and fibrosis remains unclear, and clinical translation currently lacks stratified intervention strategies based on biomarkers. Future research should prioritize large-scale longitudinal cohort studies, integrated multi-omics analyses, organoid models, and gut-lung chip platforms to identify key effector molecules and therapeutic targets, ultimately facilitating precise clinical interventions targeting the gut-lung axis.

## 1 Introduction

Pulmonary fibrosis (PF) is a chronic interstitial lung disease characterized by destruction of lung parenchymal architecture, excessive deposition of extracellular matrix (ECM), and subsequent irreversible decline in lung function (1, 2). Current pharmacological interventions for PF remain largely palliative, aimed at slowing disease progression rather than halting or reversing established fibrosis (3). The pathogenesis of PF is complex and involves multiple interconnected processes, including alveolar epithelial cell injury, abnormal proliferation and differentiation of fibroblasts into myofibroblasts, inflammatory cell infiltration, and immune dysregulation (4). Recently, accumulating evidence has implicated the gut-lung axis and its dysregulated communication in driving PF progression. PF encompasses a diverse spectrum of interstitial lung diseases (ILDs) with distinct etiologies, ranging from occupational exposures (e.g., silica- or asbestos-induced PF) and autoimmune conditions to progressive fibrosing ILDs. Among these, idiopathic pulmonary fibrosis (IPF) represents the most common, progressive, and lethal form (5). Considering that different fibrotic entities exhibit distinct pathogenetic mechanisms and microbial signatures, this review specifically addresses the gut-lung axis and microbial metabolites in IPF, supported by mechanistic evidence derived from widely utilized experimental models (e.g., bleomycin- and silica-induced PF).

The gut-lung axis is defined as a bidirectional communication pathway between the gastrointestinal tract and the lungs, mediated by microbiota, microbial metabolites, immune cells, and signaling molecules (6). Changes in gut microbiota composition and function can influence the respiratory system through a shared mucosal immune system, while microbial dysbiosis in the respiratory tract can modulate gastrointestinal function via immune pathways (7–9). Cross-sectional clinical studies have revealed a significant association between PF and distinct alterations in gut microbiota structure compared to healthy controls, characterized by an altered abundance of specific bacterial genera (10). However, these observational data do not clarify whether dysbiosis drives disease progression or results from it. Furthermore, substantial gut microbiota alterations have been observed in bleomycin-induced mouse models of PF, characterized by reduced microbial diversity and enrichment of specific bacterial taxa (11). Concurrently, fecal microbiota transplantation (FMT) from PF mice into healthy recipients was shown to exacerbate lung fibrosis, directly demonstrating the contributory role of gut microbiota in PF pathogenesis (12).

The gut microbiota communicates with the lungs primarily through its metabolites, which are instrumental in mediating the functions of the gut-lung axis and constitute the essential biochemical foundation of this interaction. Microbiota-derived metabolites play critical roles in various lung diseases, including PF, and have emerged as a rapidly developing research frontier in respiratory medicine in recent years (13). Therefore, the gut-lung axis and microbial metabolites represent key mechanisms linking PF. This review synthesizes recent advancements in understanding PF pathogenesis, focusing particularly on the gut-lung axis and microbial metabolites, while systematically evaluating the current therapeutic landscape.

## 2 Microbial metabolites as key mediators of the gut-lung axis

The human intestinal tract harbors a complex and diverse symbiotic microbial system, primarily comprising bacteria, fungi, viruses, archaea, and protozoa (14). Metabolites derived from gut microbiota are essential intermediaries in host-microbiota interactions. Cross-sectional studies have shown that the principal gut microbiota metabolites, including short-chain fatty acids (SCFAs), bile acids, and amino acid derivatives, can influence PF progression (10).

### 2.1 SCFAs

SCFAs are primarily produced by gut microbiota through anaerobic fermentation of undigested carbohydrates and host secretions, representing one of the most significant classes of metabolites involved in regulating diverse biological functions (15, 16). SCFAs can reduce PF risk through dual mechanisms. On the one hand, they exert local anti-fibrotic effects by inhibiting collagen deposition and fibroblast activation, thus reducing transforming growth factor-beta (TGF-*β*) production and suppressing interleukin-5 (IL-5) and interleukin-13 (IL-13) secretion by type 2 innate lymphoid cells (ILC2). On the other hand, in addition to local gut immune regulation, they modulate immune cells systemically through the circulatory system, thereby alleviating PF progression (17, 18). Butyrate, a representative SCFA, functions as a histone deacetylase inhibitor, modulating gene expression and exerting anti-inflammatory and anti-fibrotic effects (19). Furthermore, SCFAs maintain gut barrier integrity, preventing bacterial translocation and reducing entry of inflammatory mediators into systemic circulation (20). Notably, observational studies indicate a correlation between reduced SCFA levels and human PF, a finding also reported in bleomycin-induced animal models. Experimental supplementation of SCFAs has demonstrated efficacy in attenuating PF progression (19, 21). Collectively, these studies suggest that SCFAs exert multifaceted regulatory effects on PF through mechanisms involving immune modulation, epigenetic modification, and barrier function maintenance. Although SCFA supplementation has shown promise in alleviating bleomycin-induced PF, clinical evidence in human PF remains limited to cross-sectional studies. Thus, a causal link between SCFA deficiency and fibrosis progression in humans has yet to be established, and the therapeutic efficacy of SCFA supplementation for long-term, non-inflammatory-dependent fibrosis requires further clinical validation.

### 2.2 Bile acids

Bile acids are secondary metabolites derived from the transformation of primary bile acids by gut microbiota. These secondary bile acids can influence lung inflammation and fibrosis via systemic circulation. Notably, bile acids exhibit dual roles in PF, exerting both anti-fibrotic and pro-fibrotic effects, which appear dependent primarily on local concentrations and receptor environment. At low to moderate concentrations, certain bile acids, such as chenodeoxycholic acid (CDCA) and ursodeoxycholic acid (UDCA), exert protective effects by activating the farnesoid X receptor (FXR) and TGR5, thereby inhibiting inflammatory signaling and reducing fibroblast activation (22). In contrast, elevated concentrations of secondary bile acids, particularly deoxycholic acid (DCA) and lithocholic acid (LCA), can induce oxidative stress, disrupt mitochondrial function, and promote epithelial-mesenchymal transition (EMT), ultimately exacerbating fibrotic remodeling (23, 24). The underlying mechanism may involve TGF-β1 activation of downstream SMAD signaling pathways, promoting EMT, fibroblast proliferation, and excessive ECM deposition (25). Thus, the biological effects and cytotoxicity of bile acids appear concentration-dependent, necessitating careful control of their therapeutic levels. Currently, evidence supporting the pro-fibrotic effects of bile acids predominantly originates from micro-aspiration models simulating gastroesophageal reflux; however, these findings have not yet been validated in the highly heterogeneous human PF patient population.

### 2.3 Tryptophan metabolites

Tryptophan metabolites are specific indole compounds generated by gut microbiota that can modulate lung immunity and fibrosis through systemic circulation. Animal studies have demonstrated significant upregulation of L-tryptophan in lung tissue and serum samples from mice treated with bleomycin or silica (26). Furthermore, 5-methoxytryptophan (5-MTP), a metabolite of tryptophan, has been shown to improve lung function, alleviate alveolar structural damage in bleomycin-induced PF mouse models, and inhibit fibroblast proliferation and migration *in vitro* (27). This compound plays a significant role in the progression of PF. Moreover, indole-3-acetic acid, another key tryptophan metabolite, exhibits notable anti-fibrotic properties (28). These findings suggest that future interventions targeting PF should consider the coordinated regulation of tryptophan, its metabolites, and other microbial derivatives. However, it remains unclear whether intestinally derived metabolites can reach local concentrations sufficient to exert anti-fibrotic effects in human lung tissue.

### 2.4 Other microbial metabolites

Arginine and its metabolites are associated with matrix production in lung fibroblasts. Studies have demonstrated that arginylation in lung fibroblasts signals amino acid abundance to mTOR and GCN2 pathways, maintaining TGF-*β*-dependent signaling and consequently regulating PF (29). However, current research is primarily limited to TGF-β-related mechanisms, and additional pathways have yet to be identified. Urolithin A, a secondary metabolite produced by gut microbiota from dietary ellagitannins, exerts protective effects in PF, likely through inhibition of the PI3K/AKT/mTOR signaling pathway (30). The PI3K/AKT/mTOR pathway promotes fibroblast survival and fibrosis progression by phosphorylating apoptosis-related proteins (31). In addition, lipopolysaccharide (LPS) may induce aerobic glycolysis in lung fibroblasts, promoting lactate secretion and facilitating PF progression (32). This mechanism is potentially associated with LPS-induced histone H3K18 lactylation, which enhances METTL3 expression and fibroblast activation (33).

**Figure 1:**
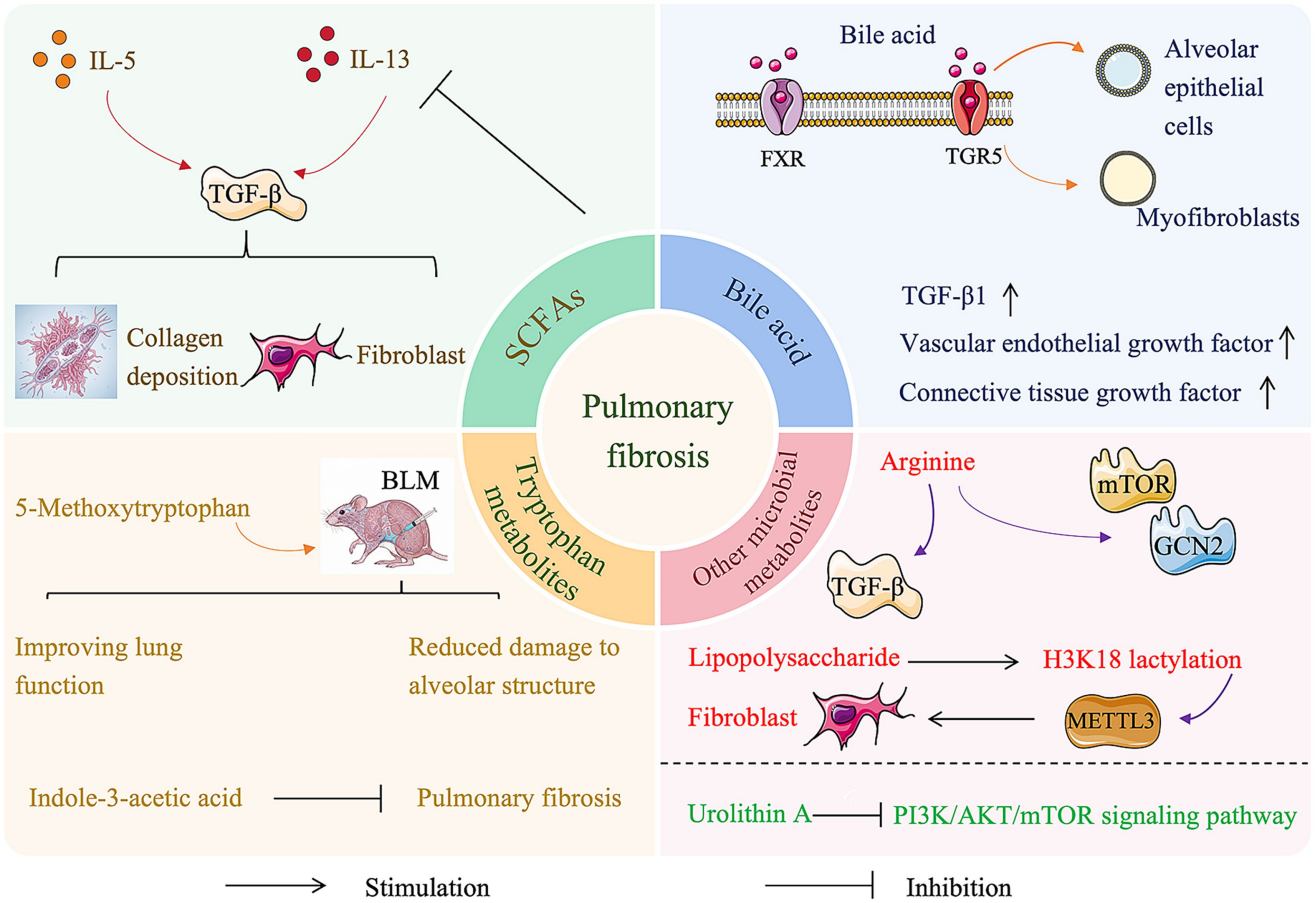
Bidirectional effects of gut-derived metabolites in PF. This figure illustrates how gut metabolites serve as mediators, directly influencing the lung microenvironment. Protective (anti-fibrotic) metabolites: SCFAs and tryptophan derivatives maintain intestinal barrier integrity, directly inhibit pulmonary fibroblast activation and collagen deposition, and decrease production of pro-fibrotic factors such as TGF-*β*. Pathogenic (pro-fibrotic) metabolites: Gut microbiota dysbiosis increases harmful substances, including LPS, elevated concentrations of secondary bile acids, and TMAO. These metabolites reach the lungs via systemic circulation, exacerbate vascular inflammation, and promote excessive fibroblast proliferation.

In summary, microbiota regulate PF via mediators such as SCFAs, LPS, bile acids, tryptophan metabolites, and arginine derivatives (Figure 1). However, existing research faces several challenges. Most orally administered SCFAs are absorbed in the intestine and undergo hepatic first-pass metabolism, complicating the attainment of effective pulmonary concentrations. LPS primarily originates from Gram-negative bacteria; complete elimination of these bacteria is neither therapeutically feasible nor ecologically desirable, and targeting interventions remains highly complex. Different bile acids exert distinct, concentration-dependent effects. For example, low concentrations of LCA may be anti-inflammatory, whereas higher concentrations may induce cytotoxicity. The therapeutic window is narrow, and systemic administration might inadvertently reach pro-fibrotic concentrations in target tissues. Moreover, the bile acid pool composition varies considerably among individuals due to differences in gut microbiota composition, dietary patterns, and genetic backgrounds, complicating intervention standardization. Regulating PF through microbial metabolites essentially represents an attempt to manage the disease by modulating the internal ecosystem. Thus, the inherent complexity of the gut microbiome presents both substantial therapeutic potential and considerable challenges for clinical standardization.

## 3 Role of the gut-lung axis in the pathogenesis of PF

### 3.1 Destruction of intestinal barrier and bacterial translocation

A healthy gut microbiota helps maintain the integrity of tight junction proteins between intestinal epithelial cells. Gut microbiota dysbiosis, particularly the excessive proliferation of Gram-negative bacteria, leads to the release of toxins that directly disrupt the epithelial barrier, resulting in increased intestinal permeability, commonly referred to as “leaky gut” (34). In animal models, gut microbiota dysbiosis has been shown to damage the intestinal barrier, facilitating the entry of bacterial products and inflammatory mediators into systemic circulation, thereby triggering or exacerbating pulmonary inflammation (35). Whether this causal sequence occurs similarly in human PF remains to be confirmed by longitudinal studies. Alterations in gut microbiota and their metabolites can induce inflammatory responses and stimulate mesenchymal cells to produce excessive ECM, which may contribute to PF pathogenesis (36). Studies have shown that following intestinal barrier disruption, the bacterial endotoxin LPS enters the bloodstream through the intestinal wall (37). It subsequently reaches the pulmonary capillary network via the portal vein or lymphatic system. Toll-like receptor 4 (TLR4) expressed on alveolar macrophages and endothelial cells recognizes LPS. Binding of LPS to TLR4 on immune cells, including alveolar macrophages and epithelial cells, activates downstream signaling pathways (31). Upon TLR4 activation, nuclear factor kappa B (NF-κB) is activated via myeloid differentiation factor 88 (MyD88)-dependent or independent pathways, inducing the release of pro-inflammatory cytokines such as TNF-*α* and IL-1*β*, ultimately leading to pulmonary inflammation and alveolar damage (38, 39). Notably, the directional migration of T helper 17 (Th17) cells between the gut and lungs and their role in promoting TGF-β secretion have primarily been demonstrated in murine models using cell-tracking techniques. In PF patients, the mechanistic link between gut-derived immune cells and PF progression remains largely speculative due to the difficulty of obtaining synchronized immune samples from the gut and lungs, and direct causal evidence is lacking.

### 3.2 Migration of immune cells to the lungs

As the site of the body’s largest lymphoid tissue reservoir, the gut plays a central role in immune regulation. Gut microbiota imbalance does not directly affect the lungs but instead modulates pulmonary inflammation through immune cell regulation in the gut (40). Specific microbial communities, such as segmented filamentous bacteria, can induce differentiation of Th17 cells. Under pathological conditions, the proportion of pro-inflammatory Th17 cells increases significantly, whereas regulatory T cells (Tregs), which maintain immune tolerance, decrease (41). Lymphocytes activated in intestinal lymphoid tissue express specific chemokine receptors, such as CCR4 and CCR6 (42). During pulmonary inflammation, corresponding ligands are released, which promote the directed migration of intestinal-derived Th17 cells expressing CCR6 to the lungs (43, 44). These migrated Th17 cells further recruit neutrophils through the secretion of cytokines such as IL-17A and induce fibroblasts to produce TGF-*β*, thereby exacerbating pulmonary inflammation and fibrosis (45–49).

### 3.3 Regulation of pulmonary inflammation by intestinal metabolites

Gut microbiota play a crucial regulatory role in the initiation and progression of PF through their metabolites. This constitutes the core mechanism of the gut-lung axis: metabolic molecules originating from the gut enter systemic circulation, directly or indirectly reshaping the pulmonary immune microenvironment, influencing epithelial cells and fibroblasts, and determining whether tissue repair or fibrosis ensues following injury.

Protective metabolites are primarily produced by abundant symbiotic bacteria, notably SCFAs and indole derivatives. These metabolites inhibit fibrosis via multiple mechanisms. Butyrate promotes the differentiation of regulatory T cells, thereby suppressing excessive inflammatory responses, restoring immune tolerance, and preventing fibroblast-to-myofibroblast differentiation (50–53). Indole compounds mitigate PF by inhibiting fibroblast proliferation and differentiation (54). Conversely, when microbial dysbiosis occurs and intestinal barrier integrity is compromised, harmful metabolites are excessively produced, driving the fibrotic process. Dysregulated bile acid metabolism can disrupt FXR signaling in the lungs, induce oxidative stress, and exacerbate lung injury (55). Trimethylamine-N-oxide (TMAO), identified as a key metabolite in PF progression, promotes NLRP3 inflammasome activation, aggravates vascular inflammation, induces endothelial dysfunction, and contributes to fibrosis (56). Additionally, the role of polyamines is subtype- and disease-stage-dependent. Although polyamines are essential for epithelial repair, they may also promote fibroblast proliferation (57), potentially driving aberrant repair processes. Specifically, polyamines form a pro-fibrotic positive feedback loop in PF through calcium-sensing receptors (CaSR): TGF-β1 stimulates polyamine synthesis and release from lung fibroblasts; subsequently, polyamines, acting as CaSR agonists, activate this receptor and further amplify TGF-β1-induced pro-fibrotic factor expression, creating a vicious cycle (58). However, spermidine, a specific polyamine subtype, can reduce inflammation and collagen deposition in bleomycin-induced PF mouse models (59). The pleiotropic complexity of polyamine metabolism, encompassing numerous enzymes, transporters, and regulatory feedback mechanisms, complicates selective inhibition of pro-fibrotic pathways without impairing essential repair functions. Future research efforts should prioritize subtype-selective modulators, stage-specific intervention windows, and non-invasive imaging or metabolomic biomarkers to enable precise targeting of polyamine pathways in PF.

In two mouse models of PF induced by bleomycin and silica dust, researchers conducted 16S rDNA sequencing and untargeted metabolomic analyses on mouse fecal samples (60). Seven intestinal bacterial taxa and nine metabolites were notably representative. Although these indicators strongly correlated with classical fibrosis markers (such as hydroxyproline, type I collagen, and fibronectin), this association alone does not confirm a causal mechanism underlying matrix deposition. Moreover, functional annotation analysis using the Clusters of Orthologous Groups (COG) method to predict bacterial functions related to PF revealed that altered gut microbiota primarily involve biological processes including cell membrane synthesis, carbohydrate transport and metabolism, gene transcription, inorganic ion transport, and coenzyme transport and metabolism (61). The aforementioned research provides a foundation for validating the reliability of specific gut microbiota and metabolites as biomarkers for PF progression and offers novel evidence supporting gut-lung regulatory interactions in PF.

### 3.4 Effects of lung injury on intestinal function

Although considerable research has addressed the unidirectional influence of gut microbiota on PF, the reciprocal effects on the intestine following irreversible PF should not be overlooked. In patients with PF, the incidence of gastrointestinal symptoms such as gastroesophageal reflux, abdominal distension, and weight loss is remarkably high, reflecting pathological and physiological effects of lung injury on intestinal function (62).

PF induces thickening of the alveolar-capillary membrane, causing impaired gas exchange and resulting in chronic systemic hypoxemia (63). Intestinal epithelial cells are highly sensitive to hypoxia. Persistent hypoxia can downregulate the expression of tight junction proteins in intestinal epithelial cells, increasing intestinal mucosal permeability (64). Notably, hypoxia-inducible factor-1α (HIF-1α) exerts protective effects initially, but its chronic activation may induce pathological angiogenesis and inflammatory responses in the intestine (65, 66). In addition, PF progression is often associated with pronounced immune activation. Damaged lung tissue releases abundant pro-inflammatory cytokines, including TNF-*α*, IL-6, and IL-1β, into systemic circulation (67). These inflammatory mediators enter intestinal microcirculation, activating intestinal macrophages, triggering local sterile inflammation, and disrupting intestinal homeostasis (68). Studies have demonstrated migration of lung-derived immune cells to the intestine.

In addition, pulmonary infections may exacerbate intestinal dysfunction. In healthy lungs, the microbiota maintains relative stability during inhalation and exhalation processes (69). In lung disease, airway microbiota equilibrium is disrupted. When bacterial proliferation exceeds the airway’s microbial clearance capacity, lung fibroblast dysfunction occurs, mucus secretion increases, and bacterial migration is enhanced, thereby elevating densities of certain gut-originating microorganisms in the lung (70). A metagenomic sequencing study analyzing gut microbiota alterations showed significantly lower microbial diversity in a pneumonia-induced sepsis group compared to healthy controls, and further microbiota reduction following antibiotic treatment (12). This mechanism may involve activation of the MAPK signaling pathway in intestinal tissue cells, thereby enhancing inflammatory responses (71). Furthermore, lung-colonizing microbial communities can exert significant biological functions by modulating the immune system (72). Notably, common microbiota alterations in mouse models of PF include decreased abundance of Bacteroidetes and increased Firmicutes abundance. Such dysbiosis correlates positively with heightened systemic inflammation (73). These lung injury-induced microbiota changes reduce SCFA production, further exacerbating intestinal injury and perpetuating a vicious cycle (74). Collectively, these findings suggest that lung injury can substantially alter gut microbiota composition.

**Figure 2:**
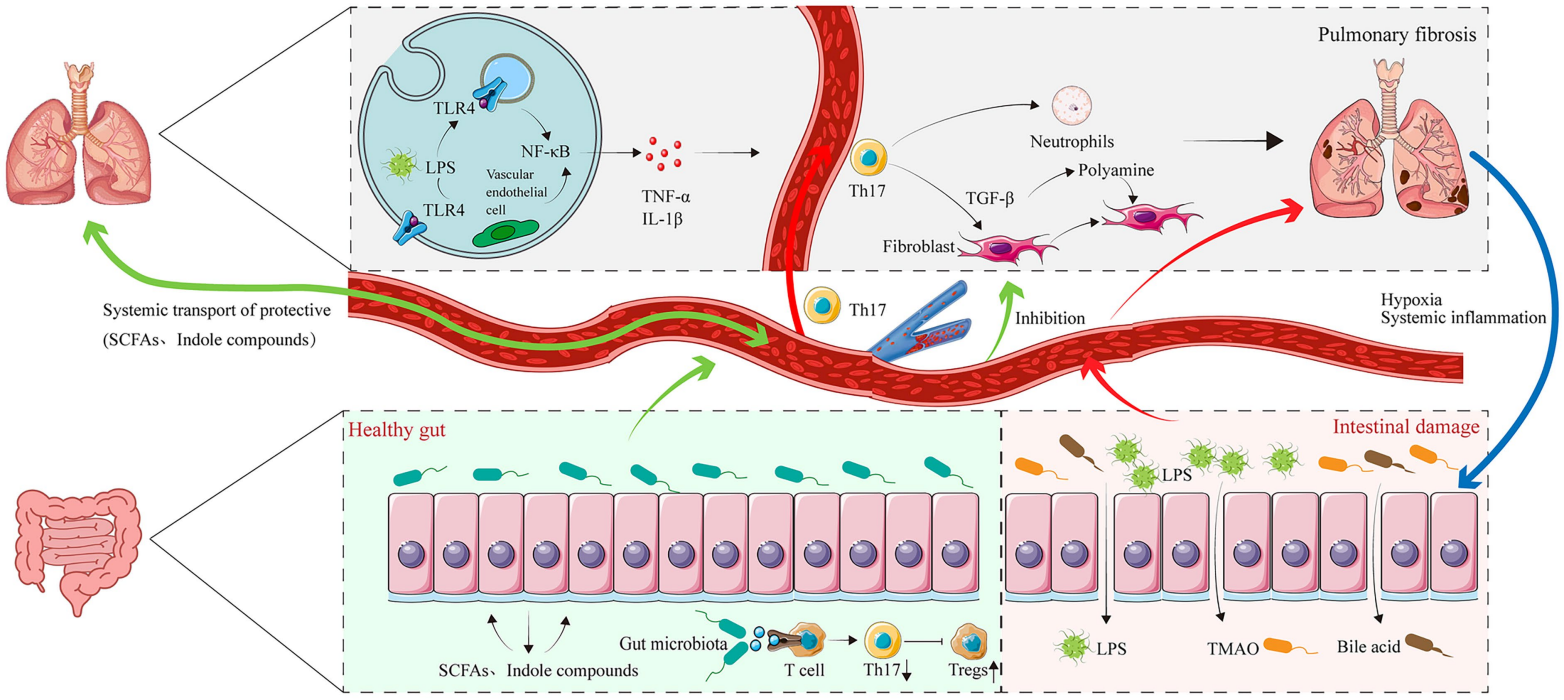
Bidirectional regulation of the gut-lung axis via metabolic signals and immune cell communication in PF. The figure illustrates three core mechanistic pathways linking gut dysbiosis to lung fibrosis. (1) Protective pathway: A healthy gut microbiota produces beneficial metabolites, including SCFAs and indole compounds. These enter the circulation (blood functions solely as a transport carrier) and systemically inhibit pulmonary fibroblast activation, reduce TGF-β production, and promote regulatory T cell differentiation, thereby counteracting fibrosis. (2) Pathogenic pathway: Gut dysbiosis disrupts intestinal epithelial integrity, enabling translocation of LPS, TMAO, and secondary bile acids into the bloodstream. These pathogenic metabolites reach the pulmonary vasculature, where LPS activates TLR4 on endothelial cells and alveolar macrophages, triggering NF-κB signaling and the release of pro-inflammatory cytokines (TNF-α, IL-1β), which exacerbate alveolar damage and fibroblast activation. (3) Fibrosis-induced hypoxia and systemic inflammation damage the intestinal barrier and exacerbate gut dysbiosis, establishing a vicious bi-directional cycle.

**Figure 3:**
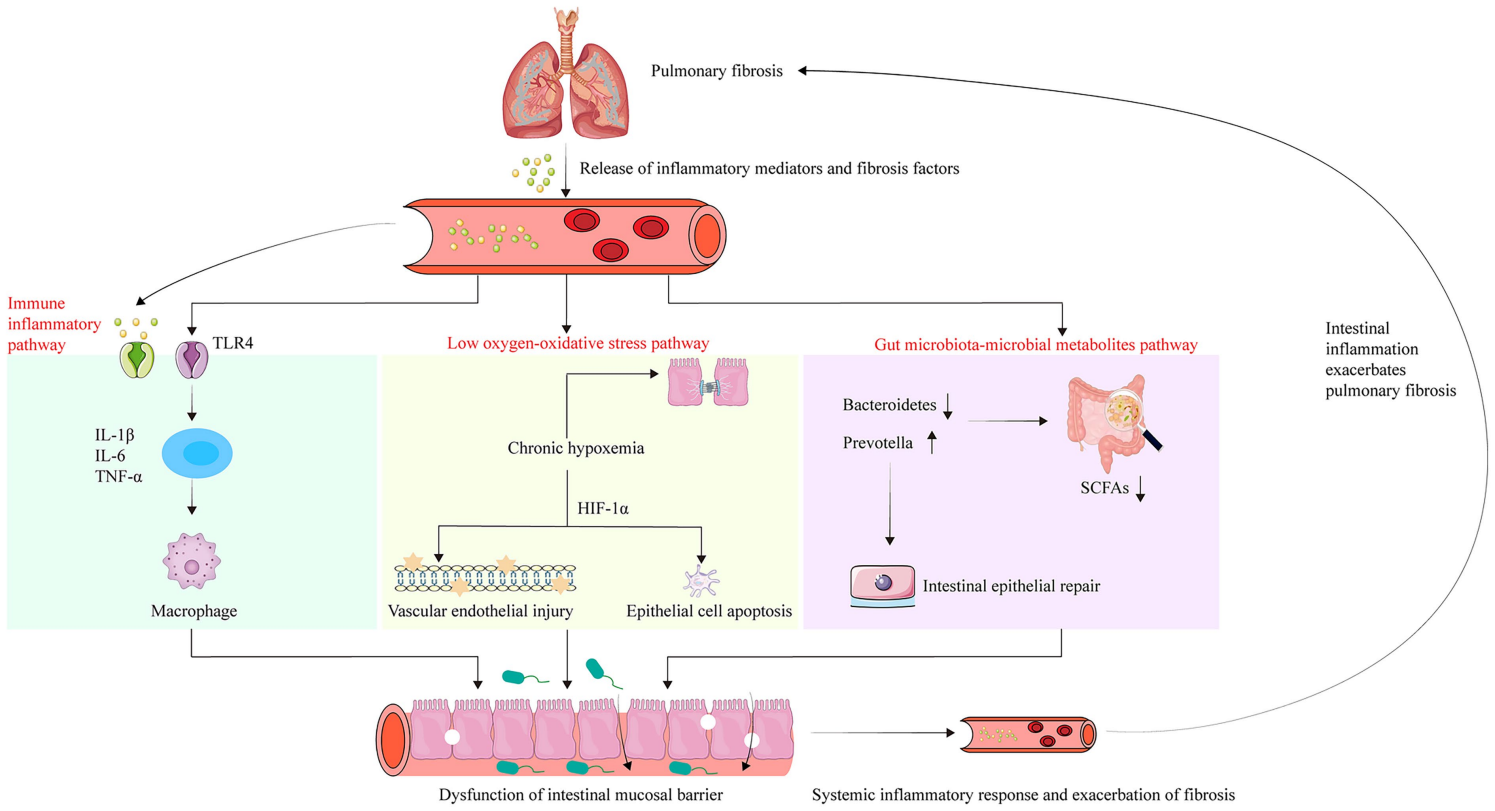
This figure highlights three major mechanisms by which lung injury perpetuates intestinal damage, creating a vicious cycle: (1) Hypoxia and stress: Reduced respiratory function in PF leads to systemic chronic hypoxia, directly compromising the intestinal mucosal barrier, which is highly oxygen-sensitive. (2) Dissemination of inflammatory cytokines: Lung injury releases substantial inflammatory mediators (e.g., TNF-α, IL-6) into systemic circulation, triggering localized intestinal inflammation. (3) Microbiota and metabolite imbalance: Lung disease decreases beneficial gut bacteria such as Bacteroides, significantly reducing beneficial metabolite production. This impairs intestinal self-repair mechanisms and further accelerates systemic fibrosis progression.

In summary, intestinal dysbiosis can disrupt barrier integrity and reduce beneficial metabolites, replacing beneficial signals with harmful substances. This process triggers systemic inflammation and immune dysfunction, altering the pulmonary microenvironment, specifically through epithelial damage, immune imbalance, and fibroblast activation, ultimately promoting PF progression (Figure 2). Conversely, hypoxia, immune dysfunction, and microbial metabolic disturbances associated with lung disease further exacerbate gut microbiota dysbiosis, forming a bidirectional pathological cycle between the gut and lungs (Figure 3). This reciprocal interaction provides a robust theoretical foundation for understanding and intervening in PF from a holistic systems biology perspective.

## 4 Targeted therapeutic approaches focusing on the gut-lung axis and microbial metabolites

### 4.1 FMT

FMT involves transferring microbial communities from a healthy donor to a recipient, serving as a potent research method for exploring causal relationships (75). Although effective for establishing causality in preclinical models, FMT has not yet advanced to standard clinical therapy for human PF. Experimental studies involving transplantation of fecal microbiota from healthy mice into PF-model mice demonstrated that FMT reshapes the gut microbiota, alleviates pulmonary inflammation and fibrosis, and prolongs survival (76). Moreover, antibiotic-treated mice subjected to bleomycin-induced fibrosis exhibited an anti-fibrotic phenotype following FMT (77). It is critical to acknowledge that the reversal observed with FMT in the bleomycin model may partly reflect the model’s inherent spontaneous remission potential. Given the progressive and irreversible nature of human IPF, prolonged survival observed in animal studies may not translate into therapeutic benefits for advanced IPF patients. Currently, no registered clinical trials specifically evaluate FMT in PF patients. Key limitations include the absence of standardized donor selection criteria, optimal dosing regimens, and administration routes tailored specifically to fibrotic lung diseases. Furthermore, most PF patients are elderly and often exposed to antibiotics and immunosuppressants, potentially impairing FMT engraftment efficacy and increasing safety risks, including bacteremia. Future clinical trials must establish donor screening protocols, define responder-stratification biomarkers, and incorporate comprehensive long-term safety monitoring.

The application of FMT in PF treatment represents an emerging and promising research direction, but it remains in an early exploratory stage, characterized by notable limitations and numerous research gaps. Firstly, its mechanism of action is not yet fully understood. Currently, there is no consensus on whether FMT can reliably restore specific levels of SCFAs in PF patients or how these metabolites traverse the intestinal barrier and precisely target lung lesions. In IPF patients with immune dysregulation, introducing new microbiota may provoke inflammatory cytokine storms or exacerbate autoimmune responses rather than producing straightforward anti-inflammatory effects. Furthermore, antifibrotic medications such as nintedanib and pirfenidone frequently cause significant gastrointestinal side effects, directly harming gut microbiota (78). If microbiota dysbiosis results from medication use or hypoxia, simply restoring microbiota via FMT may not reverse established honeycomb-like fibrotic structures in the lungs. Secondly, clinical evidence is lacking. Existing PF studies predominantly utilize the bleomycin-induced mouse model, which mainly represents fibrosis following acute inflammation. This model is typically self-limiting, short-term, and does not adequately replicate the chronic, progressive, and irreversible nature of human PF, which spans several years. Thus, although FMT can alleviate acute inflammation in mice over short durations, it may not effectively reverse long-term collagen deposition and scar formation in humans. Thirdly, significant safety concerns and long-term risks remain. PF patients often concurrently use immunosuppressants or high-dose corticosteroids (79). Under immunosuppressed conditions, even probiotics introduced by FMT may translocate, potentially leading to bacteremia or sepsis. Given the chronic nature of PF, patients may require prolonged and repeated FMT. The long-term consequences of frequent microbial interventions on host immunity remain unclear. Additionally, antibiotic usage history among PF patients represents a critical confounding factor (80). Many PF patients frequently receive antibiotics due to recurrent lung infections, and their gut microbiota often predominantly consist of antibiotic-resistant bacteria. Consequently, baseline variability greatly impacts FMT engraftment success and treatment responses, complicating statistical analyses in clinical trials. In addition, PF patients are typically frail and elderly, adding further complexity to treatment planning (81).

### 4.2 Probiotics and dietary interventions

In recent years, dietary interventions and probiotics have gradually emerged as promising potential therapies for PF. Studies indicate that folic acid supplementation significantly reduces elevated homocysteine (Hcy) concentrations in lung tissue and effectively alleviates fibrotic lesions in mice, elevating folic acid beyond its traditional nutritional role to that of a potential therapeutic agent (82). Direct supplementation with probiotics represents a straightforward approach for modulating gut microbiota composition. Specific probiotic strains reshape microbiota balance through competitive exclusion, enhancement of intestinal barrier integrity, and modulation of host immune responses. These regulatory effects can increase intestinal concentrations of beneficial metabolites, such as SCFAs, indirectly exerting anti-inflammatory and anti-fibrotic effects and mitigating PF progression (83, 84). The use of prebiotics constitutes an indirect strategy for promoting beneficial bacterial growth. Prebiotics typically comprise dietary components indigestible by the host but selectively utilized by beneficial gut bacteria (85). By providing exclusive nutrients for beneficial bacteria, prebiotics stimulate their proliferation, enhancing SCFA production and release into circulation, thereby ameliorating lung inflammation (86). A high-fiber diet offers abundant fermentation substrates for gut microbiota, promoting sustained endogenous SCFA production. Dietary fiber intake correlates with elevated SCFA levels, benefiting gut health, systematically suppressing inflammation, and demonstrating protective effects against PF in animal models (34).

Current clinical evidence remains limited to small-scale pilot studies or observational cohorts. No large randomized controlled trials have demonstrated improvements in lung function or fibrosis progression following probiotic supplementation in PF patients. A critical knowledge gap concerns whether orally administered probiotics generate detectable and functionally relevant metabolite concentrations in bronchoalveolar lavage fluid. Additionally, inter-individual variability in baseline microbiota composition, concurrent medications (particularly antibiotics and antifibrotics), and the absence of validated surrogate endpoints collectively hinder clinical translation. Future studies should prioritize strain selection based on metabolic capabilities, utilize encapsulation technologies to enhance probiotic viability, and adopt adaptive trial designs to accommodate patient heterogeneity.

### 4.3 Traditional Chinese Medicine (TCM) interventions on gut microbiota and metabolites

Accumulating preclinical evidence suggests that TCM shows potential in modulating PF progression by regulating gut microbiota and associated metabolites. Chinese herbal formulas, individual herbs, and active components may correct intestinal dysbiosis by increasing beneficial bacteria, such as Lactobacillus and Bifidobacterium, and reducing harmful bacteria (87–89). This regulatory effect helps restore intestinal barrier integrity, decrease the translocation of pro-inflammatory factors such as LPS, and subsequently alleviate pulmonary inflammation and fibrosis (90, 91). TCM components also promote the production of beneficial microbial metabolites such as SCFAs (92), which exhibit anti-fibrotic properties through mechanisms including inhibition of inflammation, modulation of immune cell functions, and reduction of oxidative stress. Furthermore, TCM can influence metabolic pathways involving bile acids and tryptophan, further modulating host immune responses and inflammation (93). In addition, TCM formulas can synergistically regulate gut microbiota and address multiple aspects of PF pathology (Table 1). Moreover, several individual TCM-derived active compounds have been identified to alleviate PF through the gut-lung axis (Table 2).

**Table 1:** The treatment of PF with TCM formula.

Intervention	Mechanism	Conclusion	References
Bu-Fei-Huo-Xue capsule	Lachnospiraceae NK4A136_ group, Bacteroidetes, and *Helicobacter pylori* decreased; the relative abundance of Candidatus_Saccharimonas and Romboutsia increased	Relieve PF by regulating gut microbiota	(95)
Jiawei Buyang Huanwu Decoction	Positive effects on changing fecal microbiota to varying degrees	Improving IPF by regulating host metabolic pathways through gut microbiota regulation	(96)
Qingwen Gupi decoction	Regulating arachidonicarachidonic acid metabolism, glycerophospholipid metabolism, and phenylalanine metabolism to exert anti fibrotic effects	Reversing bleomycin induced intestinal dysbiosis and alleviating PF	(97)
Qi-Long-Tian capsule	Improved the level of SCFAs; reduced LPS in the colon; increased the relative abundance of Bacteroidetes; reduced the relative abundance of Clostridium	Intervene in PF by regulating the different genera of gut microbiota, repairing the intestinal mucosal barrier, and reducing the entry of LPS into the bloodstream	(98)
Xuanfei Baidu decoction	Upregulation of ZO-1, Claudin-1, Occludin, and VE Cadherin protein expression; reverse these chaotic bacteria and immune microenvironments, among which Akkermansia is an important bacterium that affects the downstream systemic IFN-γ expression of the STAT1/STAT3 axis; upregulation of IFN-γ and p-STAT1 production, downregulation of p-STAT3	Regulating gut lung crosstalk to improve BLM induced IPF	(99)
Yi-Fei-Tong-Bi Decoction	Changes in abundance of induced Alloprevotella, unclassified Muribaculaceae, and Lachnospiraceae NK4A136 group	Regulating gut microbiota to alleviate BLM induced PF	(100)

**Table 2:** TCM monomers targeting the gut-lung axis for PF.

TCM monomer	Source herb	Chemical structure	Model	Gut microbiota/metabolites	Signaling pathway	Anti-pulmonary fibrosis mechanism	References
20(S)-protopanaxadiol	*Panax ginseng*	C_30_H_52_O_3_	*In vivo*: BLM induced PF	Regulated gut microbiota composition (specific taxa not detailed).	AMPK/STING signaling pathway	Directly acting on the lungs or regulating the gut microbiota.	(101)
Berberine	*Coptis chinensis*	C_20_H_18_NO_4_^+^	In vivo: BLM induced PF	No specified	PPAR-γ/HGF signaling pathway	Promote the activation of PPAR-γ in colon fibroblasts, induce HGF expression, and allow HGF to enter the circulatory system, exerting anti fibrotic effects in the lungs.	(102)
Cryptotanshinone	*Salvia miltiorrhiza Bunge*	C_19_H_20_O_3_	In vivo: Radiation induced PF	Gut microbiota: Enterorhabdus, Akkermansia, Erysipelatocostridium; Gut metabolite: bile acid	Activated FXR signaling pathway	Inhibit the process of epithelial mesenchymal transition and suppress inflammation, thereby reducing the deposition of extracellular matrix in PF	(103)
Heterophyllin B	*Pseudostellaria heterophylla*	C_40_H_58_N_8_O_8_	In vivo: BLM-induced PF; *In vitro*: MLE-12, A549 cells	Gut microbiota: muribaculum intestinale; Gut metabolite: 3-hydroxybutyric acid	IDO1/Ferroptosis signaling pathway	Relieve fusion by eliminating gut microbiota and metabolism, targeting IDO1 therapy	(73)

Despite promising preclinical findings, the clinical translation of TCM formulations targeting the gut-lung axis in PF faces significant challenges. Firstly, TCM formulas are inherently complex, multi-component botanical systems, complicating standardization efforts necessary for ensuring batch-to-batch consistency and pharmacological reproducibility. Secondly, navigating rigorous regulatory pathways for clinical approval of multi-target botanical preparations requires highly standardized pharmacological and toxicological profiling, which is difficult to achieve with traditional decoctions. Future research should prioritize improving experimental adaptability to clinical practice by integrating multi-omics approaches and artificial intelligence to construct comprehensive, dynamic network maps and identify key therapeutic targets. For instance, novel delivery systems such as nanoparticles have been explored for targeted transport of therapeutic molecules (e.g., siRNA) or metabolites directly to the lungs or intestines, effectively treating PF while reducing side effects (94). In addition, identifying predictive biomarkers to facilitate patient stratification could enhance clinical trial precision and therapeutic efficacy.

## 5 Conclusions and perspectives

Cross-sectional studies have consistently identified associations between gut dysbiosis and PF. However, it remains unclear whether these microbial alterations are drivers or consequences of the disease. Gut microbiota dysbiosis contributes to pulmonary inflammation, and microbial metabolites regulate mechanisms underlying PF progression; conversely, lung injury also significantly affects gut health. Although promising results have emerged from therapies targeting the gut-lung axis, effectiveness remains unproven in rigorous clinical trials. A critical limitation is that most mechanistic evidence derives from animal models. While human studies consistently demonstrate strong correlations between gut dysbiosis and PF, causal relationships have yet to be firmly established. It remains plausible that observed dysbiosis in patients may result secondarily from chronic hypoxia, dietary changes, or pharmacological interventions rather than representing a primary driver of PF.

Firstly, the causal relationships remain uncertain. Although existing research has identified dysbiosis and metabolic changes associated with PF, precisely distinguishing disease drivers from consequences remains challenging. Future research should employ large-scale longitudinal cohort studies to dynamically track associations between microbiota and disease progression. Notably, integrated multi-omics analyses focusing on microbial functional pathways and their key effector molecules are essential. At the mechanistic level, studies should clarify how specific metabolites regulate core fibrotic signaling pathways by targeting critical immune cell populations, such as pulmonary alveolar macrophages and regulatory T cells.

Secondly, clinical translation faces substantial practical obstacles. Although current animal models offer valuable mechanistic insights into early inflammatory responses and subsequent fibrotic signaling, they fail to accurately replicate clinical realities of PF. For instance, the bleomycin-induced model primarily involves acute injury-driven inflammation, typically self-limiting, contrasting sharply with chronic, progressive, and irreversible fibrosis observed in human PF. Therefore, therapeutic successes observed in these models should be interpreted cautiously. A significant limitation is the heavy reliance on acute injury models, such as those induced by bleomycin or silica. These models predominantly reflect self-limited, inflammation-driven fibrosis, inadequately capturing the chronic, progressive, and irreversible characteristics of human PF. Furthermore, translating findings across distinct PF subtypes requires caution, as their genetic architectures, immune landscapes, and microbiome interactions may differ considerably. Future translational research should explicitly stratify PF subtypes and prioritize human-derived data to verify whether microbiota-mediated mechanisms are universally applicable or subtype-specific.

Furthermore, the diagnosis of PF primarily relies on high-resolution computed tomography (CT) and histopathology, with limited availability of biomarkers for predicting disease progression or treatment response. Currently, SCFAs are regarded as metabolites with the highest potential for clinical translation. Considering the safety concerns associated with FMT, such as triggering inflammatory storms or autoimmune reactions in immunocompromised patients, recent clinical trials suggest that high-fiber dietary interventions have a more favorable risk–benefit profile. Future interventions should prioritize patients with early-stage PF and implement precision clinical trials stratified by biomarkers (e.g., baseline fecal SCFA levels or intestinal permeability indicators). Additionally, specific metabolite profiles in serum, urine, or exhaled breath should be evaluated as potential markers of disease activity and treatment response. To overcome limitations associated with bleomycin- or silica-induced animal models, which inadequately replicate chronic, irreversible fibrotic processes, researchers should adopt advanced *in vitro* systems, such as gut-lung chip models and patient-derived organoids, to validate the local efficacy of metabolites in human tissues. This approach is essential for bridging the significant gap between oral administration of therapies and effective PF treatment. Moreover, future research should employ single-cell and spatial multi-omics techniques to elucidate precise molecular targets and spatial dynamics of gut-derived signals within the complex pulmonary cellular networks.

In summary, progressing the gut-lung axis field from observational findings to clinical application demands a paradigm shift toward mechanistic precision, human-relevant models, and stratified intervention strategies. By integrating multi-omics analyses, organoid technologies, and biomarker-guided experimental designs, the transformative therapeutic potential of targeting the gut-lung axis in PF can be realized.

## Glossary

PF: Pulmonary fibrosis
ECM: Extracellular matrix
EMT: Epithelial-mesenchymal transition
SCFAs: Short-chain fatty acids
TGF-β: Transforming growth factor-beta
ILDs: Interstitial lung diseases
TGF-β1: Transforming growth factor-beta 1
IL-5: Interleukin-5
IL-13: Interleukin-13
ILC2: Type 2 innate lymphoid cells
5-MTP: 5-Methoxytryptophan
LPS: Lipopolysaccharide
COG: Clusters of orthologous groups
CDCA: Chenodeoxycholic acid
UDCA: Ursodeoxycholic acid
LCA: Lithocholic acid
TMAO: Trimethylamine-N-oxide
Th17: T helper cell 17
Tregs: Regulatory T cells
FXR: Farnesol X receptor
IPF: Idiopathic pulmonary fibrosis
HIF-1α: Hypoxia inducible factor-1α
FMT: Fecal microbiota transplantation
Hcy: High homocysteine
MyD88: Myeloid differentiation factor 88
CT: Computed tomography
